# Transformation to Industrial Artificial Intelligence and Workers' Mental Health: Evidence From China

**DOI:** 10.3389/fpubh.2022.881827

**Published:** 2022-05-24

**Authors:** Siying Yang, Kouming Liu, JiaHui Gai, Xiaogang He

**Affiliations:** ^1^Centre for China Public Sector Economy Research, Jilin University, Changchun, China; ^2^School of Economics, Jilin University, Changchun, China; ^3^Institute of Industrial Economics, Jiangxi University of Finance and Economics, Nanchang, China

**Keywords:** mental health, work intensity, wage income, transformation to industrial artificial intelligence, workers

## Abstract

This study matches data from the China Family Panel Studies (CFPS) with data on the transformation to industrial artificial intelligence (AI) in cities to explore the effect of this transformation on workers' mental health and its underlying mechanisms in China. The findings show the following (1). The transformation to industrial AI effectively alleviates multiple mental health problems and improves workers' mental health (2). Work intensity and wage income play an intermediary role in the relationship between the industrial AI transformation and workers' mental health (3). Potential endogeneity problems in the relationship between industrial AI and workers' mental health are considered, and robustness tests are conducted (including changing the dependent variables, independent variables and regression models). The main results and impact mechanisms remain robust and reliable. This study extends the research on the relationship between industrial AI and workers' health, which has important theoretical implications. Additionally, based on the Chinese context, this research has important implications for the current AI transformation in developing countries. Transition economies with labor shortages can achieve a win-win situation by promoting industrial AI to fill the labor gap and improve workers' mental health.

## Introduction

Artificial intelligence (AI) is an important force driving economic and social development, and it is a strategic field in which various countries compete. As the largest developing country in the world, China is currently undergoing an unprecedented process of transformation to AI, especially in the industrial field. The application of AI technologies such as industrial robots is becoming increasingly extensive, and human society is accelerating toward a new stage of high automation. The impact of the transformation to industrial AI on society is far-reaching (1), and it has become a focus of current academic research. Findings on the social impact of this transformation are mixed. On the one hand, the application of AI technology not only brings new products and services but also improves production efficiency and product quality, which play a significant role in promoting social welfare and meeting people's material and spiritual needs (2, 3). During the transformation to industrial AI, robots take the place of people to engage in dangerous work, preventing workers' exposure to hazardous working environments, which can protect occupational safety and reduce occupational injuries (4). However, on the other hand, the substitution effect of AI robots for human labor has aroused public concern about this transformation (5). For example, the application of emerging technologies such as AI increases social learning pressure and anxiety about the use of new technology (6). In addition, some studies have found that the mismatch between the application requests for industrial robots and workers' skills has not only failed to reduce occupational injuries but also led to more frequent and serious occupational injuries in emerging economies (7, 8).

However, existing studies ignore the changes in workers' mental health in the context of the transformation to industrial AI. The application of new technology has an important impact on workers' mental health (9). For example, Borle et al. (10) found that high-intensity digital work inhibited workers' mental health. We aim to clarify the impact of this transformation on workers' mental health. In the past decade, the transformation to industrial AI has led to profound changes in the labor market, directly affecting employment opportunities, methods and income (11). Undoubtedly, these changes have affected workers' mental health. For example, based on cross-border data, Cords and Prettner (12) found that this transformation led to increased unemployment and decreased wages for low-skilled workers. Jung and Lim (13) found that the application of industrial robots suppressed employment and reduced average wages. Unemployment and low income levels are important sources of workers' stress and poor mental health (14, 15). Moreover, the application of industrial robots causes technophobia and leads to negative emotions among workers, who may easily become groups with mental health problems such as anxiety and depression (16). From this viewpoint, the transformation to industrial AI may increase workers' mental health problems. However, these studies ignore an important issue; that is, this transformation may be in line with the times. For example, in a country with a serious shortage of industrial labor, the transformation to industrial AI can alleviate the labor gap faced by enterprises and reduce labor intensity (17). Furthermore, this transformation means an improvement in labor productivity, which increases workers' income level (18). As mentioned above, increased income is a favorable factor for workers' mental health.

The analysis above shows that the impact of the transformation to industrial AI on workers' mental health is uncertain (19). We explore the impact of this transformation on workers' mental health in China. The reason why we take China as the research object is that it allows us to clarify the impact of this transformation on workers' mental health when the transformation conforms to the times. We believe that the transformation to industrial AI is in line with the times in China for the following main reasons. On the one hand, China is currently facing a labor shortage, which is an important factor in promoting this transformation. China is the world's manufacturing factory, and the huge scale of its manufacturing industry involves an enormous demand for labor (20). However, factors such as the aging population and the low skill level of workers contribute to China's labor shortage (21). On the other hand, China has the economic basis and technical conditions for the transformation to industrial AI (22). In recent years, the speed of this transformation in China has surpassed that in almost any other country in the world, and the quantity of industrial robots imported ranks first in the world (3). Meanwhile, industrial AI technology is developing rapidly in China (23). Therefore, the transformation to industrial AI conforms to the Chinese reality. Furthermore, China is a transition country subject to widespread global attention. Exploring the impact of the transformation to industrial AI on workers' mental health in China is of referential significance for other transition economies to promote high-quality industrial AI (24).

Based on the above analysis, we measure the penetration rate of urban robots based on data disclosed by the International Robotics Alliance, which is regarded as a measure of the level the transformation to industrial AI in cities. We match China Family Panel Studies (CFPS) data with data on this transformation at the city level to conduct the empirical analysis. The regression results show that China's transformation to industrial AI effectively alleviates a variety of workers' mental health problems, and robustness tests based on various methods support this conclusion. Thus, we find that in a large transition economy such as China's, if the transformation to industrial AI is in line with the times, then it may help alleviate workers' mental health problems instead of causing fear of technology. Moreover, through a mediating effect model, we find that industrial AI may improve workers' mental health by increasing their wage income and reducing working hours. This research answers the questions of not only whether but also how the transformation to industrial AI affects workers' mental health, which has crucial theoretical and practical significance.

## Methods

### Data

The data used in this paper are mainly from the International Federation of Robotics (IFR) and the nationwide CFPS conducted by the China Social Science Research Center of Peking University. The data contain information on the installation of industrial robots in 50 countries from 1993 to 2018, and the industries involved are from the following six categories: agriculture, forestry, animal husbandry and fishery; mining; manufacturing; electricity, heat, gas and water production and supply; construction; and education. We use the industrial enterprise module from the Second National Economic Census data to calculate the number of people employed in different industries in each prefecture-level city (region, autonomous prefecture, and league). We measure the density of industrial robot installation in each city combined with the IFR data to measure the level of the transformation to industrial AI in the city.

CFPS is a national, comprehensive, and highly authoritative large-scale social tracking survey project that collects data from individuals, families, and communities through face-to-face or telephone interviews and off-site follow-up interviews. It leads to changes China's social, economic, demographic, educational, and health conditions and provides a public policy formulation and academic research database. The project is conducted once every 2 years, and five rounds of surveys have been conducted: in 2010, 2012, 2014, 2016, and 2018. A community questionnaire database, a household questionnaire database, an adult questionnaire database, and a pediatric questionnaire database have been established. This paper focuses on the impact of robot use on workers' mental health, uses 2014 survey data, and retains only employed individuals. We chose the survey data from the 2014 wave because there are differences in the way mental health is measured in the CFPS questionnaires for each year. In addition, China started to use robots on a large scale after 2013. After further eliminating observations with missing variables, the final valid sample size is 7,731.

### Variable Definition

#### Explained Variables

This paper focuses on the impact of the AI industrial transformation on workers' mental health, and the dependent variable is workers' mental health. The “Partial Behavior and Mental State Measurement” module of the CFPS Individual Questionnaire asked respondents about six aspects of their mental state in the past month: “How often do you feel emotionally frustrated, depressed, and unable to do anything uplifting?”, “How often are you nervous?”, “How often do you feel restless and have trouble staying calm?”, “How often do you feel hopeless about the future?”, “How often do you find it difficult to do anything?”, and “How often do you think life is meaningless?” There were 5 answer options: 1. Almost every day; 2. Often; 3. Half of the time; 4. Sometimes; 5. Never. The survey reflects workers' psychological state; the larger the value is, the better the psychological state.

#### Core Explanatory Variables

This paper's core explanatory variable is the transformation to industrial AI, measured by the density of industrial robot installations at the city level. We mainly use the IFR dataset and the Second National Economic Census industrial enterprise data to calculate the industrial robot installation density. Since the IFR data contain only industrial robot installations at the industry level and city-level industrial robot installations are not available, we draw on Acemoglu and Restrepo (5) to calculate the robot installation density with the Bartik instrumental variable to represent the intensity of the impact of robot technology. First, we match the IFR data with China's Second Economic Census data to obtain data at the industry level. Then, we select a base year to calculate the weights of robot density by industry for each city in China. We calculate the city-level industrial robot installation density accordingly. The specific calculation is as follows.


(1) $$density_{ct}=\sum_{j}\frac{robot_{jt}}{emp_{j,t=2008}}*\frac{emp_{c,j,t=2008}}{emp_{c,t=2008}}$$

where $\frac{robot_{jt}}{emp_{j,t=2008}}$ is the ratio of the stock of robots used in industry j per 10,000 employees, and $\frac{emp_{c,j,t=2008}}{emp_{c,t=2008}}$ is the ratio of employees in industry j to all employees in city c in 2008.

#### Control Variables

Referring to the existing literature, this paper controls for a series of variables that may affect employees' mental health, including employees' age and age squared; employees' gender, with 1 for male and 0 for female; whether employees have spouses, with 1 for married or cohabiting and 0 for unmarried, divorced or widowed; employees' household status, with 1 for non-agricultural and 0 for agricultural; employees' education level, measured by the number of years of education; household size, defined by the number of people sharing daily activities; household elderly dependency ratio, measured by the proportion of people over the age of 60 years within the number of household members; household child dependency ratio, measured by the proportion of children under the age of 16 years within the number of household members; whether the household is entrepreneurial, with a value of 1 if a household member had been self-employed or started a private business in the past year; household financial status, measured by household income per capita; and household debt status, measured by total household debt. Both household average income and total indebtedness are treated logarithmically. The paper also includes city-level control variables, including urban GDP, urban wage, industrial structure, and unemployment rate. Table 1 shows the descriptive statistics for each variable.

**Table 1:** Descriptive statistics.

**Variables**	**Observations**	**Average value**	**Standard deviation**	**Minimum value**	**Maximum value**
Cannot cheer up	7,731	4.267	0.858	1	5
Mental tension	7,731	4.414	0.836	1	5
Restless	7,731	4.558	0.746	1	5
No hope	7,731	4.730	0.644	1	5
Feel that life is difficult	7,731	4.550	0.736	1	5
Meaninglessness	7,731	4.763	0.594	1	5
Robot	7,731	7.686	3.163	2.378	18.540
Age	7,731	39.220	12.51	16	83
Male	7,731	0.585	0.493	0	1
With spouse	7,731	0.806	0.395	0	1
Non-agricultural household	7,731	0.446	0.497	0	1
Years of education	7,731	10.030	3.924	0	22
Family size	7,731	4.184	1.786	1	17
Household elderly dependency ratio	7,731	0.111	0.196	0	1
Household child dependency ratio	7,731	0.138	0.156	0	0.714
Home-based business	7,731	0.064	0.246	0	1
Household income per capita	7,731	9.580	0.826	5.122	11.320
Total household liabilities	7,731	3.517	5.137	0	12.950
GDP per capita	7,731	58.100	31.55	10.170	146.5
Wage per capita	7,731	10.860	0.282	10.390	11.430
Industry structure level	7,731	0.945	0.367	0.262	2.950
Unemployment rate	7,731	0.008	0.006	0.001	0.029

#### Model Settings

Given that the purpose of this study is to examine the impact of the transformation to industrial AI on workers' mental health, the following benchmark regression model is set.


(2) $$\begin{array}{r} \begin{array}{c} Mentalhealth_{ic}=\alpha +\gamma robot_{c}+X_{ic}\beta +\varepsilon_{i} \end{array} \end{array}$$

In the above equation, *i* represents an individual worker and c represents the city where the individual is located. The dependent variable *Mentalhealth*_*ic*_ denotes the individual's mental health status; the core explanatory variable *robot*_*c*_ denotes the level of the transformation to industrial AI in the employee's city. The coefficient γ reflects the marginal impact of the transformation to industrial AI on the individual's mental health, and *X*_*ic*_ is a series control variables related to individuals, families, and cities that may affect the mental health of workers. This paper uses clustered robust standard errors at the individual level.

## Empirical Results

### Baseline Estimates

Table 2 presents the ordinary least squares (OLS) regression estimation results of the impact of the transformation to industrial AI on workers' mental health. Columns (1)–(6) contain the six individual aspects reflecting workers' psychological health. After adding individual-, family- and city-level control variables, we find that the estimated coefficient of this transformation is always positive and significant at the 1% level, indicating that this transformation in cities significantly improves workers' mental health. Thus, in contrast to studies such as Borle et al. (10), we do not find evidence of negative effects of emerging technology adoption on workers' mental health. In contrast, we find that in a large manufacturing country such as China, the transformation to industrial AI significantly improves workers' mental health and effectively alleviates multiple psychological problems. This suggests that this transformation in China is timely in its impact on workers' mental health, which may be because it improves workers' income level and reduces labor intensity. We will test this mechanism in a follow-up study.

**Table 2:** Impact of AI transformation on workers' mental health: baseline estimates.

	**(1)**	**(2)**	**(3)**	**(4)**	**(5)**	**(6)**
	**Depress**	**Nervous**	**Calm**	**Hope**	**Difficulty**	**Meaningful**
Robot	0.0201^***^	0.0106^***^	0.0101^***^	0.0083^***^	0.0076^**^	0.0084^***^
	(0.0035)	(0.0035)	(0.0030)	(0.0026)	(0.0030)	(0.0024)
Age	−0.0072	−0.0122^**^	−0.0059	−0.0067	−0.0094^**^	−0.0129^***^
	(0.0055)	(0.0051)	(0.0047)	(0.0042)	(0.0048)	(0.0038)
Age^2^	0.0140^**^	0.0193^***^	0.0085	0.0083^*^	0.0137^**^	0.0143^***^
	(0.0065)	(0.0059)	(0.0055)	(0.0050)	(0.0056)	(0.0045)
Male	0.1223^***^	0.1065^***^	0.1020^***^	0.0826^***^	0.0680^***^	0.1070^***^
	(0.0203)	(0.0199)	(0.0179)	(0.0157)	(0.0176)	(0.0145)
Spouse	0.0507	0.0247	0.0295	0.0737^***^	0.0760^***^	0.0706^***^
	(0.0321)	(0.0297)	(0.0266)	(0.0252)	(0.0286)	(0.0222)
Non-agricultural	−0.0490^**^	−0.0520^**^	−0.0467^**^	−0.0503^***^	−0.0408^**^	−0.0165
	(0.0228)	(0.0221)	(0.0201)	(0.0177)	(0.0197)	(0.0162)
Educ_year	0.0017	−0.0019	0.0103^***^	0.0077^***^	0.0086^***^	0.0103^***^
	(0.0030)	(0.0029)	(0.0026)	(0.0024)	(0.0027)	(0.0022)
Family size	0.0113^*^	−0.0007	0.0062	0.0138^***^	0.0097^*^	0.0132^***^
	(0.0060)	(0.0062)	(0.0055)	(0.0043)	(0.0051)	(0.0043)
Elderly_ratio	−0.0656	−0.0621	−0.0157	−0.0781^*^	−0.0596	−0.0757^*^
	(0.0563)	(0.0548)	(0.0522)	(0.0465)	(0.0515)	(0.0434)
Child_ratio	−0.0861	0.0291	−0.0127	−0.0034	0.0609	0.0277
	(0.0711)	(0.0687)	(0.0617)	(0.0529)	(0.0602)	(0.0484)
Selfemploy_family	−0.0130	−0.0059	−0.0175	0.0372	−0.0602^*^	−0.0070
	(0.0389)	(0.0383)	(0.0337)	(0.0256)	(0.0310)	(0.0269)
L_wincome_per	0.0598^***^	0.0426^***^	0.0568^***^	0.0537^***^	0.0819^***^	0.0609^***^
	(0.0142)	(0.0140)	(0.0123)	(0.0105)	(0.0127)	(0.0106)
L_wtotal_debts	−0.0056^***^	−0.0080^***^	−0.0083^***^	−0.0058^***^	−0.0098^***^	−0.0048^***^
	(0.0019)	(0.0019)	(0.0017)	(0.0015)	(0.0017)	(0.0014)
Pgdp	−0.0001	−0.0004	0.0007	0.0001	0.0009^*^	0.0004
	(0.0006)	(0.0005)	(0.0005)	(0.0004)	(0.0005)	(0.0004)
Lnpwage	0.0233	0.2255^***^	−0.0336	0.0184	0.0157	0.0308
	(0.0716)	(0.0705)	(0.0617)	(0.0563)	(0.0616)	(0.0491)
Industry structure	0.0074	−0.0307	0.0324	−0.0101	−0.0246	0.0061
	(0.0317)	(0.0295)	(0.0264)	(0.0247)	(0.0286)	(0.0211)
Unemp	−9.1411^***^	−9.3019^***^	−6.2199^**^	−6.4433^***^	−6.6718^***^	−6.2626^***^
	(2.9239)	(2.9241)	(2.5421)	(2.2200)	(2.4937)	(2.0623)
_cons	3.2887^***^	1.7515^**^	4.2079^***^	3.9363^***^	3.5206^***^	3.8152^***^
	(0.7471)	(0.7306)	(0.6359)	(0.5806)	(0.6401)	(0.5107)
*N*	7,731	7,731	7,731	7,731	7,731	7,731
R^2^	0.0219	0.0204	0.0181	0.0188	0.0242	0.0287

Robust standard errors are reported in parentheses. ^***^, ^**^, and ^*^ denote significance at the 1%, 5%, 10% levels, respectively.

### Instrumental Variable Estimation

Table 2 benchmark estimation results show that robot use significantly improves employee mental health. However, the results may be biased, and the setting of the baseline regression equation may involve endogeneity problems due to omitted variables and bidirectional causality. On the one hand, there may be unobserved factors that affect workers' mental health that also affect robot use, leading to the omitted variable problem; on the other hand, workers' mental health may reverse the demand for robot use, so a reverse causality problem may exist. This paper draws on Acemoglu and Restrepo (5) to mitigate the potential endogeneity problem by using industrial robot density in the US to construct the instrumental variable for the corresponding sample city as follows:


$$\begin{array}{l} robot\text{\_}IV_{ct}=\underset{j}{\sum }\frac{robo{t\text{\_}US}_{jt}}{emp_{j,t=2008}}*\frac{emp_{c,j,t=2008}}{emp_{c,t=2008}} \end{array}$$

where *robot*_*US*_*jt*_ denotes the robot use stock of industry j in the US in year t, *emp*_*c, t* = 2008_ is the employment of industry j in China in 2008, *emp*_*j, t* = 2008_ is the employment of industry j in city c in China in 2008, and emp_*emp*_*c, j, t* = 2008_ is the employment of industry j in city c in China in 2008.

Using robot density as an instrumental variable for other countries globally with industrial robot development similar to that in China is a common approach in the relevant literature. The choice of using US industrial robot data is based on the following considerations. First, before 2013, China's robot use had long relied on imports, and the US was one of its main import countries. Second, during the sample period, the development trend of industrial robot applications in the US was relatively close to that in China, and robot technology in the US led the world. Industrial robot applications in the US can reflect the AI trend and satisfy the correlation assumption. Third, there is no evidence that the application of industrial robots in the US directly affects the mental health of employees in China (who are affected only by the application of industrial robots in China), satisfying the exogeneity assumption.

In this paper, the two-stage least squares (2SLS) method is used for the instrumental variable estimation, and the regression results are presented in Table 3. Columns (1)–(6) present the regression results using the density of industrial robot stock in the US as an instrumental variable, and column (7) contains the first-stage estimation results. The regression coefficients of the instrumental variables are positive and significant, indicating that industrial robot application promotes the mental health of workers.

**Table 3:** Instrumental variable estimation.

	**(1)**	**(2)**	**(3)**	**(4)**	**(5)**	**(6)**	**7**
	**Depress**	**Nervous**	**Calm**	**Hope**	**Difficulty**	**Meaningful**	**Robot**
Robot	0.0189^***^	0.0100^*^	0.0091^*^	0.0166^***^	0.0069	0.0130^***^	
	(0.0061)	(0.0060)	(0.0053)	(0.0045)	(0.0053)	(0.0043)	
Robot-IV							13.6619^***^
							(0.2405)
Control variables	Yes	Yes	Yes	Yes	Yes	Yes	Yes
Weak identification test							3,227
*N*	6,644	6,644	6,644	6,644	6,644	6,644	6,644
*R* ^2^	0.0211	0.0204	0.0185	0.0200	0.0257	0.0286	0.5059

Clustered robust standard errors are reported in parentheses below the coefficients. ^***^, ^**^, and ^*^ denote significance at the 1%, 5%, 10% levels, respectively. Control variables are estimated as in the Table 2 benchmark.

### Robustness Test

#### Substitution of Core Explanatory Variables

Before 2013, more than 70% of China's industrial robots were imported from Japan, Europe, and North America (3). According to an IFR report in 2014, the number of new robots sold in China was 23,000 in 2012, of which Chinese suppliers produced only approximately 3,000, and this percentage was even lower before August 2012. Therefore, we use robot import data calculated from the China Customs Trade Database to measure industrial robot applications for robustness tests. Specifically, we match the imported industrial robot data from that database to importing firms and their locations to obtain the number of imported industrial robots and the total price of imports in the city. We take the logarithmic value of the above two indicators and include them in Equation (1) for estimation.

Tables 4A,B show the estimation results of replacing the core explanatory variables with the number of industrial robots imported and the total price of imports. The table shows that the coefficients of the effects of the number of imported industrial robots installed and the import price on the mental health of workers are positive and significant at the 1% level. This indicates that the transformation to industrial AI has improved the mental health of workers and that the benchmark results described above are robust.

**Table 4:** Robustness test: replacing core explanatory variables.

	**(1)**	**(2)**	**(3)**	**(4)**	**(5)**	**(6)**
	**Depress**	**Nervous**	**Calm**	**Hope**	**Difficulty**	**Meaningful**
**(A) Core explanatory variable is the number of robot imports**						
Robot_number	0.0110^***^	0.0075^**^	0.0107^***^	0.0098^***^	0.0074^***^	0.0072^***^
	(0.0032)	(0.0033)	(0.0029)	(0.0026)	(0.0028)	(0.0024)
Control variables	Yes	Yes	Yes	Yes	Yes	Yes
*N*	8,092	8,092	8,092	8,092	8,092	8,092
*R* ^2^	0.032	0.031	0.027	0.027	0.037	0.035
**(B) Core explanatory variable is robot import price**						
Robot_price	0.0033^**^	0.0029^*^	0.0043^***^	0.0039^***^	0.0033^**^	0.0031^***^
	(0.0015)	(0.0015)	(0.0013)	(0.0012)	(0.0013)	(0.0011)
Control variables	Yes	Yes	Yes	Yes	Yes	Yes
*N*	8,092	8,092	8,092	8,092	8,092	8,092
*R* ^2^	0.032	0.034	0.028	0.028	0.040	0.036

Clustered robust standard errors are reported in parentheses below the coefficients. ^***^, ^**^, and ^*^ denote significance at the 1%, 5%, 10% levels, respectively. Control variables are estimated as in the Table 2 benchmark.

#### Substitution of Dependent Variable

Above, the dependent variable employee mental health is a subjective indicator reported by respondents. To exclude the interference of measurement error, we convert the dependent variable into a 0-1 variable. The specific approach is as follows: First, the mean values of the indicators of the six dimensions of mental health are calculated separately. Second, six 0-1 variables are defined, and if the mental health value is above the mean level, the value is set as 1. At this point, the dependent variable is a 0-1 variable, and we use the binary probit model to re-estimate the impact of the transformation to industrial AI on workers' mental health. Table 5A shows the estimation results. We find that the coefficient of industrial robot penetration is still significantly positive, which is consistent with the baseline estimation results.

**Table 5:** Robustness tests: replacing the dependent variable and replacing the regression model.

	**(1)**	**(2)**	**(3)**	**(4)**	**(5)**	**(6)**
	**depress_dum**	**nervous_dum**	**calm_dum**	**hope_dum**	**difficulty_dum**	**meaningful_dum**
**(A) Binary probit model**						
Robot	0.0257^***^	0.0160^***^	0.0150^**^	0.0156^**^	0.0102^*^	0.0223^***^
	(0.0081)	(0.0057)	(0.0060)	(0.0068)	(0.0059)	(0.0071)
Control variables	Yes	Yes	Yes	Yes	Yes	Yes
*N*	7,731	7,731	7,731	7,731	7,731	7,731
**(B) Ordered probit model**						
Robot	0.0286^***^	0.0160^***^	0.0162^***^	0.0174^***^	0.0123^**^	0.0232^***^
	(0.0050)	(0.0052)	(0.0055)	(0.0064)	(0.0054)	(0.0068)
Control *v*ariables	Yes	Yes	Yes	Yes	Yes	Yes
N	7,731	7,731	7,731	7,731	7,731	7,731

Robust standard errors are reported in parentheses below the coefficients. ^***^, ^**^, and ^*^ denote significance at the 1%, 5%, 10% levels, respectively. Control variables are estimated as in the Table 2 benchmark.

#### Replace Regression Model

Since the explanatory variable in this paper, employee mental health, is an ordered category variable, it takes values in the range of 1–5. Direct estimation by OLS is likely to cause the fitted values to fall outside the valid interval and thus lead to bias in the estimated coefficients. In such cases, scholars usually use maximum likelihood estimation (MLE) to obtain consistent estimates of the coefficients of the ordered probit model. Therefore, this estimation method is used in this paper, and the results are presented in Table 5B. The estimated coefficient of industrial robot penetration is still significantly positive, indicating that the transformation of industrial AI promotes the mental health of workers.

#### Changing Clustered Standard Error

Above, we control for clustered robust standard errors at the individual level due to heteroskedasticity. Considering that there may be commonality and correlation among different workers within the same city, we cluster the standard errors to the city level and include province fixed effects. Table 6 shows the estimation results. The coefficient of industrial robot penetration remains significantly positive, again indicating the robustness of the finding that the transformation to industrial AI promotes workers' mental health.

**Table 6:** Robustness test: changing the level of clustered standard errors.

	**(1) Depress**	**(2) Nervous**	**(3) Calm**	**(4) Hope**	**(5) Difficulty**	**(6) Meaningful**
Robot	0.0242^***^	0.0145^**^	0.0163^***^	0.0084^**^	0.0140^**^	0.0096^***^
	(0.0061)	(0.0058)	(0.0054)	(0.0041)	(0.0055)	(0.0036)
Control variables	Yes	Yes	Yes	Yes	Yes	Yes
Provincial fixed effects	Yes	Yes	Yes	Yes	Yes	Yes
*N*	7,731	7,731	7,731	7,731	7,731	7,731

Robust standard errors are reported in parentheses below the coefficients. ^***^, ^**^, and ^*^ denote significance at the 1%, 5%, 10% levels, respectively. Control variables are estimated as in the Table 2 benchmark.

After the robustness tests on the above aspects, the key findings of this paper hold. It is thus clear that the transformation to industrial AI in China can effectively improve workers' mental health.

### Mechanism Analysis

The above analysis shows that robot use significantly improves workers' mental health. Then, what are the underlying mechanisms of action? It has been shown that the use of robots increases labor productivity and has complementary effects on workers in non-routine tasks, which in turn increases workers' wages and earnings (3). Meanwhile, Aghion et al. (25) found that due to business stealing effects, automated firms displace their competitors, expand their production scale and increase their productivity, which in turn have a positive impact on employee employment and wages. Lower income levels and higher work intensity are important sources of increased psychological stress and mental illness among workers (15). To test these mechanisms, the following econometric model is constructed drawing on the existing literature.


(3) $$\begin{array}{r} \begin{array}{c} Intervariable_{ij}=\beta_{0}+\beta_{1}robot_{ij}+\beta_{2}X_{ij}+\varepsilon_{i} \end{array} \end{array}$$

In the above equation, *Intervariable*_*ij*_ represents the mediating variables in this paper, denoting employee wage income and working hours. *X*_*ij*_ denotes individual- and city-level control variables. Other variables are defined similarly to those in the benchmark model (2).

The regression results are shown in Table 7, where column (1) presents the estimation results for the effect of the transformation to industrial AI on the wage income of employees. This transformation significantly increases workers' wage income. Column (2) presents the estimation results of this transformation on the working hours of workers. The transformation significantly reduces employees' working hours. The results of these two mechanism tests indicate that the use of robots significantly improves mental health, mainly by increasing wage income and decreasing working hours.

**Table 7:** Mechanism test.

	**(1)**	**(2)**
	**Wage income**	**Working hours**
Robot	0.0309^***^	−0.2708^**^
	(0.0039)	(0.1119)
Age	0.0912^***^	0.6433^***^
	(0.0065)	(0.1754)
Age^2^	−0.1135^***^	−1.0643^***^
	(0.0074)	(0.2101)
Male	0.4267^***^	5.9851^***^
	(0.0219)	(0.5800)
Spouse	0.0872^***^	−3.1904^***^
	(0.0332)	(0.8334)
Non-agricultural	0.0377	−5.0320^***^
	(0.0245)	(0.6834)
Educ_year	0.0429^***^	−0.7842^***^
	(0.0032)	(0.0890)
Pgdp	0.0005	0.0251
	(0.0006)	(0.0162)
Lnpwage	0.6319^***^	−8.1292^***^
	(0.0764)	(2.0642)
Industry structure	−0.0498	1.3566
	(0.0313)	(0.9344)
Unemp	−7.0962^**^	67.6066
	(2.9744)	(82.1810)
_cons	0.4667	131.8503^***^
	(0.7921)	(21.4466)
*N*	6443	7731
*R* ^2^	0.1843	0.0646

Clustered robust standard errors are reported in parentheses below the coefficients. ^***^, ^**^, and ^*^ denote significance at the 1%, 5%, 10% levels, respectively.

## Discussion

With the new phase of the industrial revolution, an increasing number of studies have focused on the impact of industrial robot applications on local labor markets, including the employment structure, wage levels, and employee health (24, 26). The transformation to industrial AI is also gradually changing the human mindset, but little literature has focused on the psychological changes of workers in the context of this transformation, especially changes in workers' mental health. We explored the impact of this transformation on workers' psychological health and its mechanisms of action in the Chinese context based on matched data from the Chinese Household Tracking Survey and the penetration rate of industrial robots in cities.

We found that the transformation to industrial AI significantly improves workers' mental health. Considering that endogeneity problems due to omitted variables may have confounded our findings, we further tested these findings by constructing instrumental variables using US industrial robot data, and the results indicated that the transformation to industrial AI improves workers' mental health. In addition, we conducted robustness tests based on a range of methods, and the results also affirmed that this transformation improves workers' psychological wellbeing. This finding has important theoretical implications. In the existing literature, there are two contrasting effects of industrial transformation on workers' psychological wellbeing: unemployment and low income due to this transformation suppress workers' psychological wellbeing, or high income and low labor intensity due to this transformation promote workers' psychological wellbeing (17). Our findings support the latter conjecture.

In addition, job income and work intensity are important sources of changes in workers' mental health (14, 27). Therefore, we further examined the effects of the transformation to industrial AI on workers' psychological health. We found that this transformation improves workers' psychological health by increasing their wage income and reducing their work intensity. Our study fills the gap in the research on workers' mental health in the context of the transformation to industrial AI.

This paper provides a new perspective for understanding the changes in workers' mental health during the transformation to industrial AI. By linking the use of robots at the city level to workers' mental health at the individual level, this paper improves the understanding of the relationship between technological upgrading and micro-level individual behavior. At the same time, this paper fills an important research gap in the context of AI, thus providing valuable policy implications for international comparisons. Additionally, empirical evidence from China provides reference value for other transition economies to promote high-quality industrial AI. The findings of this paper corroborate those of Cheng et al. (28), who find that China's overall view of robots has been positive, with little mention of the threat of job replacement in government documents promoting robot adoption and production. Rather than worrying about job replacement, the government emphasizes the adoption of robots as a way to address labor challenges. The conclusions of this paper suggest that the transformation to industrial AI has instead improved the mental health of workers. Thus, AI is in line with the times, and we need not be overly concerned about the disruptive effects of AI on the workforce. The findings of this paper have important practical implications for driving AI change in developing countries.

## Conclusion

This paper matches CFPS data with urban robot data provided by IFR and uses OLS regression, probit regression, ordered probit regression, and instrumental variables to study the impact of the transformation to industrial AI on workers' mental health. We found that robot use significantly influences workers' mental health, improving all six aspects of workers' mental health. The findings remained robust after a series of robustness tests, such as replacing explanatory and explained variables and replacing regression models. In addition, to avoid endogeneity problems due to omitted variables and two-way causality, the density of industrial robots in the US was used to construct the density of industrial robots at the corresponding sample city level as an instrumental variable. The conclusions showed that the benchmark results are robust and reliable. The mechanism analysis shows that robots significantly increase workers' wage income and decrease workers' working hours, verifying the mechanism effect of robots on workers' mental health levels.

With the rapid development of robotics, there is considerable literature focusing on the impact of technological progress on the labor market. However, attention to micro-level of individuals remains insufficient, and even less literature has explored workers' mental health in the context of technological change. Accordingly, this paper examines changes in workers' mental health in the context of the transformation to industrial AI in China. The paper further explores the mechanisms of the effects of robot use on workers' psychological health from the perspectives of both wage income and work intensity. This study extends the research on the relationship between industrial AI and workers' health, which has important theoretical implications. Additionally, based on the Chinese context, this paper has important implications for the current AI changes in developing countries. Transition economies with relative labor shortages can achieve a win-win situation by promoting industrial AI to fill the labor gap and improve workers' psychological health.

Of course, there are some limitations in this paper. First, the data used are cross-sectional and do not reveal long-term persistent effects of the transformation to industrial AI on workers' mental health levels. Similarly, limited by data availability, this paper measures the transformation to industrial AI at the city level, which does not accurately reflect workers' exposure to and use of industrial robots. Second, although we explored the mechanisms underlying the effects of the transformation to industrial AI on workers' mental health, we did not explore its boundary conditions in depth. Future research can further explore the mechanisms and boundary conditions of the effects of this transformation on workers' mental health at the micro-level of individuals.

## Data Availability Statement

The original contributions presented in the study are included in the article/supplementary material, further inquiries can be directed to the corresponding author/s.

## Author Contributions

SY drafted and critically revised the paper for intellectual content. KL and JG made substantial contributions to the concept and design of the work, data interpretation, and the drafting of the article. XH drafted and critically revised the paper and polished the language of the revised draft. All authors contributed to the article and approved the submitted version.

## Funding

This work was funded by the National Natural Science Foundation of China (72163016) and the Special Research Project on Labor Relations of Jilin University (2020LD004).

## Conflict of Interest

The authors declare that the research was conducted in the absence of any commercial or financial relationships that could be construed as a potential conflict of interest.

## Publisher's Note

All claims expressed in this article are solely those of the authors and do not necessarily represent those of their affiliated organizations, or those of the publisher, the editors and the reviewers. Any product that may be evaluated in this article, or claim that may be made by its manufacturer, is not guaranteed or endorsed by the publisher.
